# Chicago Veterinary College Banquet

**Published:** 1903-05

**Authors:** 


### CHICAGO VETERINARY COLLEGE BANQUET.

The Annual Banquet given to the Faculty and Students by the Trustees of the Chicago Veterinary College was held at the Sherman House, Wednesday evening, March 11, 1903, at 8 P.M. A sumptuous menu was served, and the tables being cleared and the cigars passed around, Professor E. L. Quitman acted as toastmaster. Professor A. H. Baker responded to the toast, "The Chicago Veterinary College." Mr. F. H. McMahon, to the toast, "Conclusions of a Senior Student." Mr. G. H. Harland, to the toast, "Conclusions of a Junior Student." W. R. Edwards, to the toast, "Aspects of the Profession in the South." On behalf of the faculty, Professor J. M. Wright responded to the toast, "The Profession, Past." Professor A. S. Alexander, to the toast, "The Profession, Present." Professor G. A. Lytle, to the toast, "The Profession, Future." Professor J. Dill Robertson, M.D., to the toast, "The Sister Profession." For the Alumni, Dr. P. Quitman responded to the toast, "The Alumni of the Chicago Veterinary College." Numerous impromptu toasts were responded to by the various members of the faculty and invited guests present. The musical programme furnished was as follows:

Song—"The Goblins," by the College quartette, Messrs. Axby, Parks, Hisgen, and Perkins. "Bedouin Love Song," Solo, by Mr. W. H. Cork, Jr. Song—"Barcarolle," by Mr. J. M. Parks. Song—"The Toreador Song," by Mr. W. H. Cork, Jr. Song—"Farmer John," by the College quartette, Messrs. Axby, Parks, Hisgen, and Perkins. Whistling Solo, by Professor G. M. Cushing. Song—"The Holy City," by Mr. J. M. Parks. Piano Solo, by Mr. Irving C. Hancock.

The dining-hall was beautifully decorated with ferns and roses.

All present had an enjoyable time, and the function was concluded with three cheers for the C. V. C.

---

